# Orthognathic Surgery Using 1-Desamino-8-D-Arginine Vasopressin (DDAVP) in a Patient With Von Willebrand Disease: A Case Report

**DOI:** 10.7759/cureus.106917

**Published:** 2026-04-12

**Authors:** Satoshi Usuda, Shin Kato, Hideyuki Shiba, Taneaki Nakagawa, Seiji Asoda

**Affiliations:** 1 Department of Dentistry and Oral Surgery, Keio University School of Medicine, Tokyo, JPN; 2 Department of Dentistry and Oral Surgery, Kawasaki Municipal Ida Hospital, Kanagawa, JPN

**Keywords:** 1-desamino-8-d-arginine vasopressin (ddavp), bilateral sagittal split ramus osteotomy, lefort 1 osteotomy, orthognathic surgery, von willebrand disease

## Abstract

Von Willebrand disease (vWD) is a bleeding disorder with autosomal-dominant inheritance caused by quantitative and qualitative abnormalities in von Willebrand factor (vWF), which plays a role in primary hemostasis. In such cases, it is necessary to evaluate hemostatic function prior to surgery and take appropriate measures to manage bleeding. There are few reports on orthognathic surgery, which is a highly invasive procedure in the oral and maxillofacial regions, and all of them use blood products. We report a case of orthognathic surgery using 1-desamino-8-D-arginine vasopressin (DDAVP), a synthetic antidiuretic hormone, in a patient with vWD for jaw deformity. We report a case of orthognathic surgery using DDAVP, a synthetic antidiuretic hormone, in a patient with vWD for jaw deformity. A 26-year-old Japanese female visited another clinic with a chief complaint of mandibular prognathism and started preoperative orthodontic treatment for jaw deformity. Preoperative screening and examinations revealed prolonged APTT and decreased coagulation factor activity. The patient was referred to the hematology department at our hospital and diagnosed with type Ⅰ vWD (quantitative reduction in vWF). The patient was referred to our department for orthognathic surgery, and LeFort I osteotomy and bilateral sagittal split ramus osteotomy were planned to improve occlusion. The patient underwent a challenge test using intravenous injection of DDAVP in the hematology department to establish a response to DDAVP prior to surgery. As a result, an increase in vWD antigen levels and vWF activity was confirmed, and surgery was performed using DDAVP. There were no perioperative complications, and the patient’s perioperative course was uneventful.

## Introduction

von Willebrand disease (vWD) is a bleeding disorder with autosomal-dominant inheritance caused by quantitative and qualitative abnormalities in von Willebrand factor (vWF) [1], which plays a role in primary hemostasis. It is often encountered in the oral and maxillofacial regions during surgical procedures.

vWD has two functions: it acts as an adhesion molecule, causing platelets to adhere and aggregate at the site of bleeding, forming a platelet thrombus (primary hemostasis), and it binds to and stabilizes factor VIII (secondary hemostasis) [2]. The other binds to and stabilizes factor VIII (secondary hemostasis). vWD is classified into type I (quantitative reduction of vWF), type II (qualitative abnormality of vWF), and type III (complete absence of vWF), according to the quantitative or qualitative abnormality of vWF. Type I vWD, including our case, is a relatively mild symptom that occurs frequently (70-80%) [3,4].

vWD is typically diagnosed based on the patient’s medical history and laboratory examination, including screening tests (e.g., evaluation of bleeding time, activated partial thromboplastin time (APTT), and platelet count); confirmatory tests (e.g., evaluation of vWF function using ristocetin cofactor assay (vWF:ristocetin cofactor (RCo)), vWF protein concentration immunoassay (vWF:antigen (Ag)), and FVIII coagulation assay (FVIII:C)); and specialized tests for characterization of vWD type (e.g., structural assays such as vWF multimer analysis and vWF propeptide (vWFpp), and functional assays such as vWF binding to platelet GPIb, collagen (vWF:CB), or FVIII (vWF:FVIIIB) [5].

Surgical interventions present a critical hemostatic challenge in patients with vWD, necessitating careful perioperative management to minimize the risk of bleeding. Previous studies have reported minor oral surgical procedures and tooth extractions in patients with vWD. Still, evidence on orthodontic surgery in this population is limited [6], and all cases involved blood products. However, the use of blood products carries risks of side effects, including the development of factor VIII inhibitors, liver dysfunction due to hepatitis virus infection, and the onset of acquired immunodeficiency syndrome (AIDS). We herein report a case of orthognathic surgery with 1-desamino-8-D-arginine vasopressin (DDAVP) in a patient with vWD.

## Case presentation

**Figure 1:**
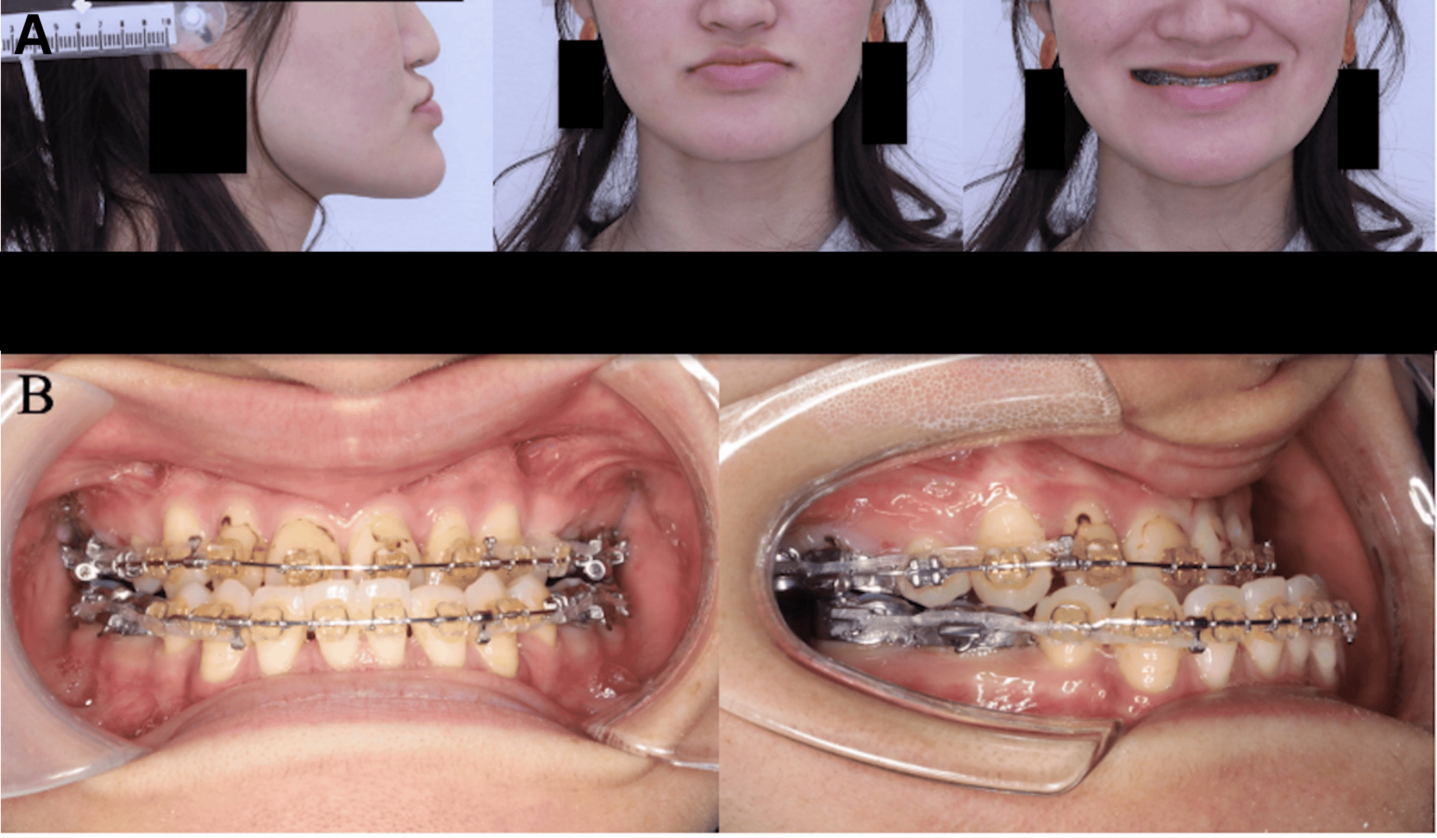
Preoperative photographs (A) Facial photographs. The frontal view was asymmetrical, with a leftward deviation of the mandible and a downturned right mouth corner, and the lateral view showed a concave type. (B) Intraoral photographs. The intraoral findings revealed an angle class III malocclusion bilaterally, with an overjet of 9.7 mm and an overbite of 3 mm.

A 26-year-old Japanese female visited another hospital with the chief complaint of a protruding lower jaw and underwent preoperative orthodontic treatment, as she was diagnosed with skeletal mandibular prognathism. Preoperative screening and examinations revealed prolonged APTT and decreased coagulation factor activity. The patient was referred to our hospital's hematology department and diagnosed with type I vWD. In Japan, multimer analysis and genetic testing for vWD are not covered by the national health insurance system and are therefore not routinely performed in clinical settings. Accordingly, the diagnosis was made based on clinical findings, prolonged APTT, and decreased vWF antigen and activity levels, in line with standard diagnostic practice in Japan. The patient was referred to our department because the referring hospital was unable to perform orthognathic surgery on a patient with a bleeding disorder. The patient was 169.5 cm tall, weighed 51 kg, and had a good general condition. There were no characteristic symptoms of vWD, such as petechiae, epistaxis, or bleeding from the mouth, and there was no abnormality in family history. Regarding facial appearance, the frontal view was asymmetrical, with a leftward deviation of the mandible and a downturned right mouth corner, and the lateral view showed a concave type (Figure 1A). The intraoral findings revealed an angle class III malocclusion bilaterally, with an overjet of 9.7 mm and an overbite of 3 mm (Figure 1B).

**Figure 2:**
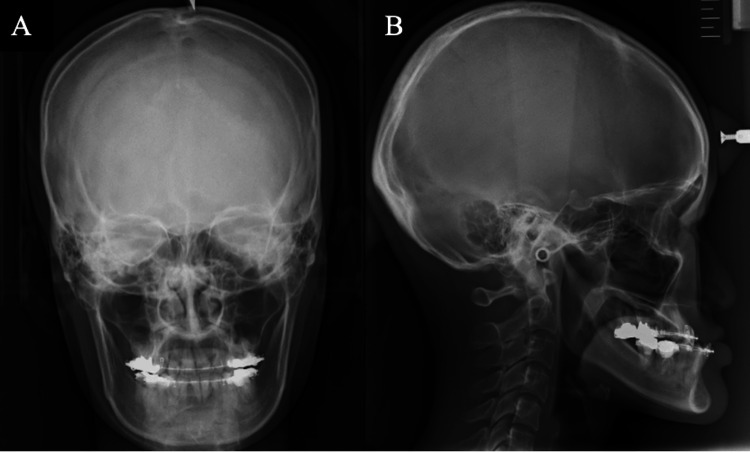
Preoperative radiographs (A) Posteroanterior cephalometric radiographs. (B) Lateral cephalometric radiographs. Based on the cephalometric radiograph taken at the initial examination, the patient was diagnosed with mandibular prognathism.

Based on the cephalometric radiograph taken at the initial examination, the patient was diagnosed with mandibular prognathism (Figure 2A-2B), and we planned to perform LeFort I osteotomy and bilateral sagittal split ramus osteotomy (BSSO) to improve the occlusion.

The blood test showed a prolonged APTT (40.7 seconds) and low coagulation factor VIII (61%), vWD antigen (37%), and vWF activity (30%), but there were no other obvious abnormal parameters (Table 1).

**Table 1:** Blood test results at the first visit WBC: white blood cell, RBC: red blood cell, HGB: hemoglobin, HCT: hematocrit, PLT: platelet, vWF: von Willebrand factor, vWD: von Willebrand disease, PT: prothrombin time, APTT: activated partial thromboplastin time

Testes	Case	Reference ranges	Unit
				WBC	4.5	3.5-8.5	10^3^/μL
				RBC	3.9	3.7-4.9	10^4^/μL
HGB	12.4	11.5-15	g/dL
HCT	37.1	35-45	%
PLT	300	150-350	10^3^/μL
PT	12.1	11-13.5	Seconds
APTT	40.7	23-36	Seconds
Coagulation factor VIII	61	78-165	%
vWD antigen	37	50-155	%
vWF activity (RCo)	30	60-170	%

**Figure 3:**
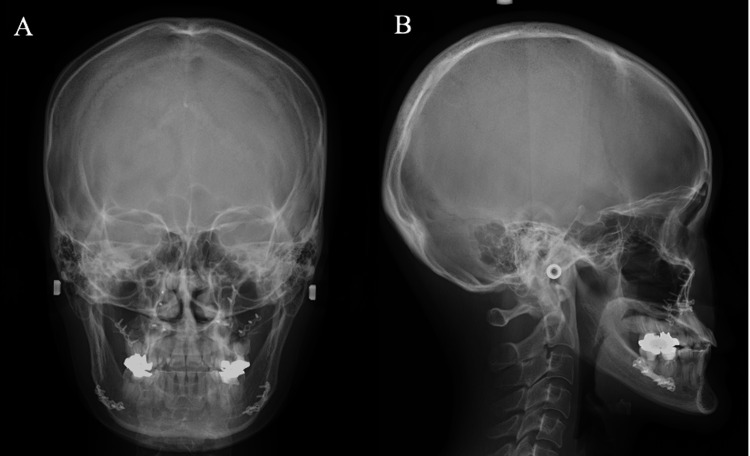
Postoperative radiographs (A) Posteroanterior cephalometric radiographs. (B) Lateral cephalometric radiographs. LeFort I osteotomy and BSSO were performed under general anesthesia. Both the upper and lower osteotomized segments were plated using titanium osteosynthesis.

After explaining the risks of orthognathic surgery in vWD, the patient wished to undergo surgery to improve her occlusion. Accordingly, the patient underwent a challenge test with intravenous DDAVP (0.4 μg/kg) in the hematology department to establish a response prior to surgery. As a result, the vWD antigen increased from 48% to 89%, and the vWF activity increased from 33% to 95%. Therefore, it was decided that surgery could be performed with DDAVP, and DDAVP was administered at a dose of 0.4 μg/kg 30 minutes prior to surgery. Autologous blood (800 mL) was collected prior to surgery, and LeFort I osteotomy and BSSO were performed under general anesthesia (Figure 3A-3B). Both the upper and lower osteotomized segments were plated using titanium osteosynthesis. The operative time was 289 minutes, and the operative blood loss was 310 mL. On postoperative day 1, a blood test showed vWF activity of 96%, and there was no postoperative bleeding.

Additionally, 400 mL of autologous blood was transfused back into the patient on the day of surgery and the following day. Both DDAVP (0.4 μg/kg) and tranexamic acid (10 mg/kg) were administered intravenously 30 minutes prior to surgery. Postoperatively, both drugs were administered every 12 hours until postoperative day 3 to ensure adequate hemostasis during the perioperative period. No abnormal bleeding was observed during hospitalization, and the drain was removed on postoperative day 5 after an additional intravenous dose of DDAVP. To minimize the risk of hyponatremia, we carefully monitored electrolyte balance by checking serum sodium levels through intraoperative blood gas analysis and postoperative blood tests.

**Figure 4:**
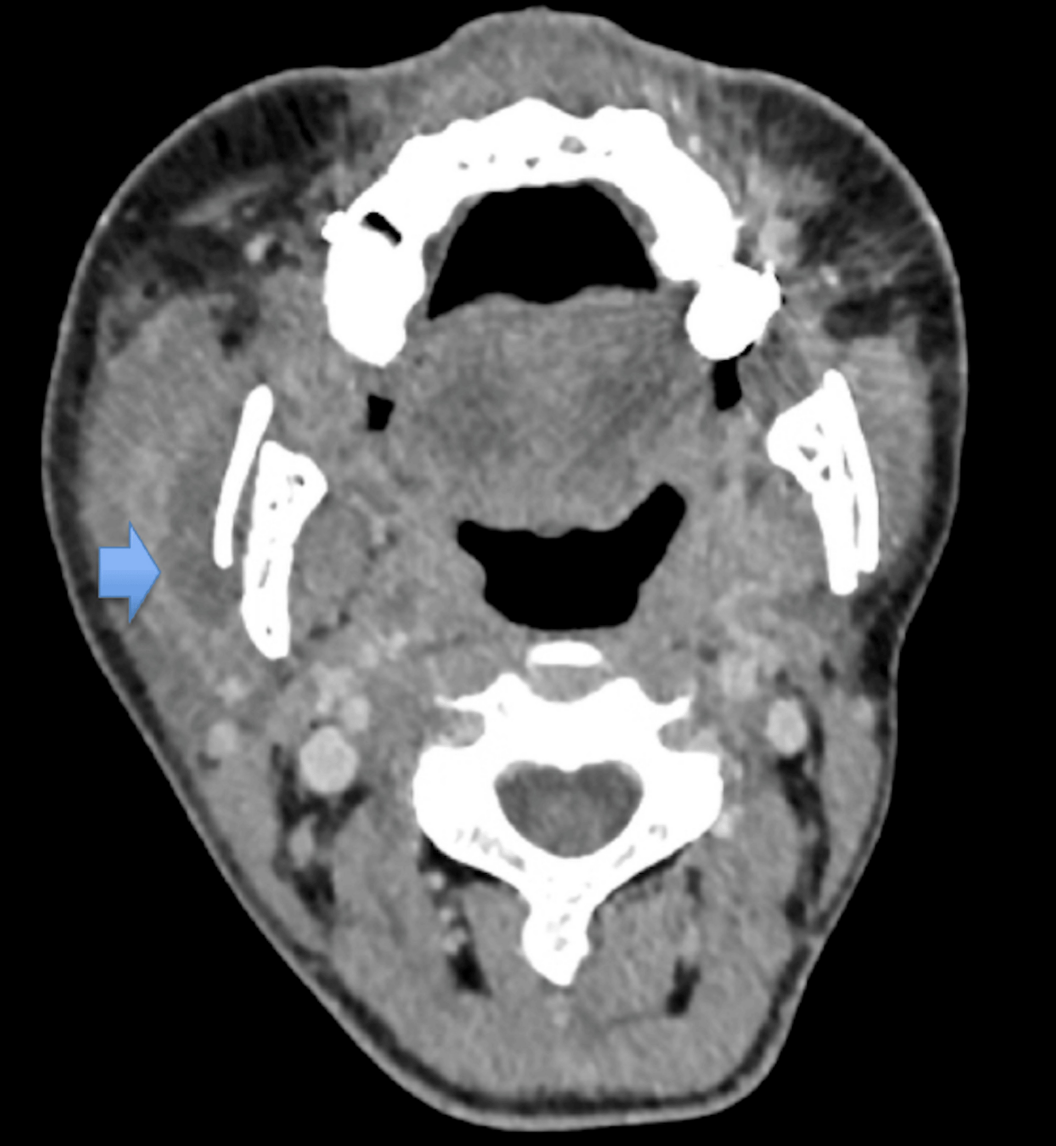
CT image Swelling of the right cheek and a fever of 38.0°C were observed on postoperative day 30. CT images showed a low-density area between the right mandible and the masseter muscle, suggesting fluid accumulation (blue arrows). CT: computed tomography

Additionally, the patient was advised to avoid excessive fluid intake. The patient was discharged on postoperative day 14. Subsequently, routine follow-up was performed on an outpatient basis. However, swelling of the right cheek and a fever of 38.0°C were observed on postoperative day 30. Blood tests showed elevated CRP (4.83 mg/dL) and WBC (11300 μL). CT images showed a low-density area between the right mandible and the masseter muscle, suggesting fluid accumulation (Figure 4). As an infection of the hematoma was suspected, the patient was re-hospitalized and treated with sulbactam/ampicillin (4.5 g/day). The swelling subsided after antibiotic administration, and the patient was discharged after a blood test on rehospitalization day 7, which showed improved inflammatory findings, with CRP (0.4 mg/dL) and WBC (5200 μL). We have not observed a recurrence of swelling since then.

## Discussion

vWD is the most common hereditary hemorrhagic disease in humans and is a congenital coagulation disorder that causes bleeding due to quantitative and qualitative abnormalities in vWF [1]. The estimated prevalence of vWD in the general population is approximately 1-2%, although only a small proportion of individuals are clinically symptomatic [7]. WF has two functions: it acts as an adhesion molecule, causing platelets to adhere and aggregate at the site of bleeding, forming a platelet thrombus (primary hemostasis), and it binds to and stabilizes factor VIII (secondary hemostasis) [2]. The other binds to and stabilizes factor VIII (secondary hemostasis). vWD is classified into type I (quantitative reduction of vWF), type II (qualitative abnormality of vWF), and type III (complete absence of vWF), according to the quantitative or qualitative abnormality of vWF. Type I vWD, including our case, is a relatively mild symptom that occurs frequently (70-80%) [3,4].

In the past, recombinant factor VIII concentrates containing vWF were used to manage hemostasis in patients with vWD. Still, there were problems with side effects, such as the development of factor VIII inhibitors, liver dysfunction due to hepatitis virus infection, and the development of AIDS. In contrast, DDAVP, a synthetic antidiuretic hormone, has three important advantages: (1) it is inexpensive, (2) there is no risk of infection with viruses or new pathogens, and (3) it prevents exposure to clotting factors and platelet preparations, reducing the risk of an immune response [8]. Therefore, DDAVP is now widely administered to patients with mild or moderate vWD symptoms [9]. However, DDAVP is generally not indicated for vWD types II and III because of disadvantages such as the inability to administer it repeatedly over a short period of time and the uncertainty of increased deficiency factor activity, although it may be used in selected cases of type II, depending on the subtype and individual response [10,11]. Additionally, a challenge test with intravenous DDAVP injection must be performed at least two weeks prior to surgery to confirm an increase in the deficient factor's activity [12].

There are no definite standards for perioperative management in orthognathic surgery in patients with vWD. However, it is recommended that vWF activity be maintained at 30% or more for one to five days for minor surgeries. vWF activity should be initially maintained at 100% and then at least 50% for 7-10 days for major surgeries and severe bleeding, according to the 2007 vWD guidelines from the US National Heart, Lung, and Blood Institute (NHLBI) [13]. Because repeated DDAVP administration reduces its efficacy, factor VIII concentrates containing vWF are recommended when DDAVP cannot be administered, or in cases of severe bleeding or major surgery [13]. It has been reported that orthognathic surgery is associated with a high risk of bleeding [1], and there are few reports on orthognathic surgery and perioperative management in patients with vWD. To the best of our knowledge, there have been nine reports, including our own (Table 2) [6,14-16]. Among them, six cases were suspected to be type I vWD, and five cases, excluding the present case, were treated with factor VIII products. None of the cases were managed with DDAVP alone, as in our case.

**Table 2:** A case of orthognathic surgery in patients with vWD To the best of our knowledge, there have been nine reports, including our own. Among them, six cases were suspected to be type I vWD, and five cases, excluding the present case, were treated with factor VIII products. None of the cases were managed with DDAVP alone, as in our case. In some cases, treatment details are marked as “unknown,” indicating that the original article did not provide specific information about the treatment. F: female, M: male, SSRO: sagittal split ramus osteotomy, LeFort I: Le Fort I osteotomy, DDAVP: 1-desamino-8-D-arginine vasopressin, Factor VIII: factor VIII concentrate rich in von Willebrand factor

Case no.	Year	Authors	Sex	Age	Type of vWD	Operation	Treatment	Complication	Blood loss (cc)	Operating time (min)
1	1990	Ilankovan et al. [14]	F	23	Type 1	SSRO	Factor VIII	None	Unknown	Unknown
2	1990	Ilankovan et al. [14]	F	18	Type 1	LeFort I, SSRO, genioplasty	Factor VIII	None	Unknown	Unknown
3	1992	Holtzman et al. [15]	M	22	Unknown	LeFort I, IVRO	DDAVP +Factor VIII	None	350	Unknown
4	2022	Kasahara et al. [6]	F	34	Unknown	SSRO	Unknown	Unknown	Minimal	137
5	2024	Sato et al. [16]	M	28	Type 1	SSRO	Factor VIII	None	638	238
6	2024	Sato et al. [16]	M	19	Type 3	SSRO	Factor VIII	None	834	279
7	2024	Sato et al. [16]	F	26	Type 1	LeFort I, SSRO	Factor VIII	None	554	375
8	2024	Sato et al. [16]	F	29	Type 1	SSRO	Factor VIII	None	154	216
9	2024	Reporter	F	26	Type 1	LeFort I, SSRO	DDAVP	None	310	289

The decision regarding the procedure and hemostatic management for this case was carefully reviewed with the hematologist and the referring orthodontist in accordance with the above guidelines. First, we considered performing BSSO alone because we wanted to use a minimally invasive surgical technique with a low risk of bleeding. However, the results of the preoperative surgical simulation showed that BSSO alone produced substantial posterior mandibular movement. Therefore, we decided it was necessary to move both the upper and lower jaws to achieve appropriate occlusion. Regarding hemostasis management, the patient had the relatively mild type I vWD, and the response to DDAVP was obtained in advance, with sufficient activation; therefore, we decided to administer DDAVP as the first-line treatment. In the oral and maxillofacial regions, DDAVP is used for minor-to-moderate surgery and is often administered during tooth extraction in patients with vWD [17]. However, relative to tooth extraction, Le Fort I osteotomy and BSSO are more invasive and may cause abnormal bleeding. Accordingly, we planned to administer factor VIII concentrate in the case of abnormal operative bleeding or difficult hemostasis. Fortunately, there was no abnormal bleeding during surgery. DDAVP was administered every 12 hours for three days postoperatively. Because DDAVP has a short half-life and its effect is attenuated by tachyphylaxis after repeated administration [8], it was combined with intravenous tranexamic acid as an adjuvant therapy. This therapy has been shown to effectively reduce the risk of perioperative bleeding and the need for blood transfusions in patients undergoing orthognathic surgery [18]. Moreover, we applied hypotensive anesthesia, which intentionally lowers blood pressure to 55-80 mmHg [19], to minimize operative blood loss. Although hyponatremia often occurs with the administration of DDAVP [8], no abnormalities were observed perioperatively in this case.

As a technique, we first performed a model operation using surgical simulation software (Proplan CMF®, Materialise, Leuven, Belgium) to understand the anatomical morphology of the descending palatine artery and the course of the inferior alveolar nerve in accordance with the actual bone-cutting line. Bone removal around the descending palatine artery was performed gently with an ultrasonic cutting device (Piezotome®, Hakusui Boeki, Osaka, Japan) to avoid severe bleeding due to vascular injury. Generally, the amount of bleeding for both upper and lower osteotomies is 854.8 ± 442.8 ml, and the operation time is 292.4 ± 64.7 min [20]. However, our operation was below average, with no unusual abnormalities. We usually loosen the intermaxillary elastics on postoperative day 3 to allow the patient to open their mouth a little. Still, in this case, we did not loosen the elastics until postoperative day 7 to prevent postoperative bleeding by stabilizing the wound. For postoperative pain, acetaminophen was administered to avoid the antiplatelet effects of non-steroidal anti-inflammatory drugs.

There is no evidence of the most appropriate perioperative management for preventing surgical bleeding in patients with vWD. The approach differs depending on the type of vWD and the patient's condition. Close collaboration with other departments enables the accurate diagnosis of blood disorders, and safe treatment is possible even for patients with vWD by obtaining informed consent after sufficient consideration of hemostasis, surgical, and anesthesia methods. Patient satisfaction was also assessed in this case, suggesting that the use of less-invasive DDAVP is an option in orthognathic surgery for patients with vWD. However, further case accumulation is necessary to determine the applicability and usefulness of this treatment.

## Conclusions

We performed orthognathic surgery using DDAVP in a patient with type I vWD. The use of blood products for invasive procedures in patients with vWD is useful from a hemostatic perspective, but it carries risks of infection and cost implications. It is important to accurately assess the patient's vWD status, carefully consider surgical techniques and devices, prevent surgical bleeding, and explore surgical approaches that do not rely on blood products as the first choice. This case report and literature review provide important insights into the diagnosis and treatment of this rare disease. This will help clinicians provide appropriate surgical management.
